# Youth psychopathic traits and delinquent behavior among adolescents in Saudi social observation centers: the indirect role of moral disengagement and the moderating role of cognitive empathy

**DOI:** 10.3389/fpsyg.2026.1895885

**Published:** 2026-07-29

**Authors:** Bandar Salah Almailabi

**Affiliations:** Department of Education, College of Arabic Language and Humanities, Islamic University of Madinah, Madinah, Saudi Arabia

**Keywords:** cognitive empathy, delinquency, juvenile delinquency, moral disengagement, PLS-SEM, youth psychopathic traits

## Abstract

**Introduction:**

Juvenile delinquency is associated with multiple individual-level psychological domains, but youth psychopathic traits, moral disengagement, and cognitive empathy have been less directly examined within a single theory-informed structural model among justice-involved adolescents. This study tested a cross-sectional structural model linking youth psychopathic traits, moral disengagement, cognitive empathy, and delinquent behavior among adolescents residing in social observation centers in Saudi Arabia.

**Methods:**

The sample included 171 adolescents recruited from nine social observation centers across Saudi Arabia. Youth psychopathic traits were assessed using the Youth Psychopathic Traits Inventory-Short Version, moral disengagement using the eight-item Propensity to Morally Disengage Scale, cognitive empathy using the cognitive empathy subscale of the Basic Empathy Scale, and delinquent behavior using a short self-report delinquency index. Data were analyzed using partial least-squares structural equation modeling with 5,000 bootstrap resamples.

**Results:**

Youth psychopathic traits were positively associated with moral disengagement (β = 0.302, *p* < 0.001) and delinquent behavior (β = 0.217, *p* < 0.001). Moral disengagement was positively associated with delinquent behavior (β = 0.153, *p* = 0.006) and represented a small statistically detectable indirect component of the concurrent association between youth psychopathic traits and delinquent behavior (β = 0.046, *p* = 0.030). Cognitive empathy was negatively associated with delinquent behavior (β = −0.344, *p* < 0.001). The interaction between youth psychopathic traits and cognitive empathy was significant (β = –0.175, *p* = 0.007), indicating that the positive association between youth psychopathic traits and delinquent behavior was weaker at higher levels of cognitive empathy. The model explained 36.4% of the variance in delinquent behavior.

**Discussion:**

The findings are consistent with a social-cognitive moral-regulation framing in which delinquent behavior is associated with dispositional psychopathy-related traits, moral-cognitive justification, and social-cognitive understanding. Because the data were cross-sectional, the indirect path through moral disengagement was interpreted as a statistically estimated concurrent indirect association rather than evidence of temporal mediation or a causal mechanism. The interaction involving cognitive empathy was likewise interpreted as a conditional concurrent association rather than evidence of a causal buffering effect.

## 1 Introduction

Juvenile delinquency refers to criminal or law-breaking acts committed by juveniles and is commonly examined in relation to psychopathic traits and current or future offending outcomes (Geerlings et al., 2020). Although delinquent behavior is legally defined through acts that violate formal rules, psychological research examines the individual and social factors associated with why some adolescents show greater involvement in such behavior than others. Prior work indicates that delinquency is associated with multiple domains of risk, including individual, social, and opportunity-related factors (Bobbio et al., 2020; Geerlings et al., 2020). Within this broader risk framework, individual-level psychological characteristics remain important for understanding variation in delinquent behavior during adolescence.

Within individual-level explanations of juvenile delinquency, youth psychopathic traits provide a dispositional framework for describing interpersonal, affective, and behavioral characteristics relevant to delinquent behavior. In adolescent research, these traits are commonly represented by grandiose–manipulative, callous–unemotional, and impulsive–irresponsible dimensions, reflecting interpersonal grandiosity and manipulation, callous–unemotional features, and impulsive–irresponsible behavioral tendencies (Colins et al., 2012; Gillen et al., 2019). Meta-analytic evidence indicates that psychopathic traits in juveniles are positively associated with delinquency and recidivism-related outcomes, although the magnitude of this association varies by psychopathy dimension and some study and sample characteristics (Asscher et al., 2011; Geerlings et al., 2020). This evidence provides a theoretically relevant basis for examining how psychopathy-related dispositional characteristics are associated with variation in delinquent behavior during adolescence.

Moral disengagement adds a moral-cognitive dimension to this dispositional account. In Bandura’s social-cognitive theory of moral agency, moral standards regulate conduct through anticipatory self-sanctions; however, these self-sanctions can be selectively weakened when harmful conduct is morally justified, responsibility is displaced or diffused, injurious consequences are minimized, or victims are blamed or dehumanized (Bandura et al., 1996; Bandura, 2002). Empirical work with adolescents has linked higher moral disengagement to antisocial behavior, aggression, violence, and offending-related outcomes (Hyde et al., 2010; Paciello et al., 2008; Shulman et al., 2011). Longitudinal evidence indicates that adolescents who maintain higher levels of moral disengagement across adolescence are more likely to show frequent aggressive and violent acts in late adolescence (Paciello et al., 2008). More broadly, meta-analytic evidence indicates that moral disengagement is associated with transgressive behavior in youth and is embedded within personal and environmental influences that may increase or suppress its enlistment (Luo and Bussey, 2023). Prior evidence also suggests that moral disengagement is empirically connected with callous–unemotional or psychopathy-related traits. Muratori et al. (2017) reported reciprocal longitudinal associations between moral disengagement and callous–unemotional traits in adolescents with disruptive behavior disorders, whereas Paciello et al. (2020) concluded that the relation between these affective and cognitive–moral domains is complex and may involve mediation, moderation, or reciprocal longitudinal patterns. More recent offender-sample evidence has also linked callous–unemotional traits, empathy, moral disengagement, and externalizing behavior within a correlational path model among juvenile offenders (Gomez Tabares et al., 2025). Accordingly, this evidence is best interpreted as supporting a theoretically relevant association between psychopathy-related traits and moral disengagement, not as evidence for a confirmed unidirectional developmental pathway. Moral disengagement is therefore relevant to delinquency research as a moral-cognitive process associated with the justification of harmful or rule-violating conduct.

Cognitive empathy adds a social-cognitive component to this framework because it concerns the capacity to understand another person’s emotional experience or perspective. It is distinguishable from affective empathy, which involves sharing or resonating with another person’s emotional state; this distinction is central to the Basic Empathy Scale and its cognitive–affective structure (Jolliffe and Farrington, 2006). Meta-analytic evidence indicates that lower cognitive empathy is more strongly associated with offending than lower affective empathy. Jolliffe and Farrington (2004) similarly reported that low cognitive empathy was strongly related to offending, whereas low affective empathy was weakly related. In a later meta-analysis, van Langen et al. (2014) found a stronger association for cognitive empathy (*d* = 0.43) than for affective empathy (*d* = 0.19), with effect sizes varying by study and participant characteristics, including age, sex, assessment instrument, and matching variables. Because cognitive empathy concerns perspective understanding rather than affective resonance, it is theoretically relevant as a social-cognitive capacity that may condition the association between psychopathy-related characteristics and delinquent behavior. However, direct adolescent evidence testing cognitive empathy as a moderator of the psychopathy–delinquency association remains limited; the present study therefore treats cognitive empathy as a theory-informed boundary condition rather than an established causal moderator.

The preceding literature provides a theory-informed social-cognitive moral-regulation basis for organizing these constructs. Youth psychopathic traits locate delinquency-related risk at the level of dispositional interpersonal, affective, and behavioral tendencies; moral disengagement locates it at the level of moral self-regulation, where harmful conduct can be cognitively justified and self-sanctions weakened; and cognitive empathy locates it at the level of social-cognitive understanding of others’ emotional states and perspectives (Bandura et al., 1996; Bandura, 2002; Colins et al., 2012; Jolliffe and Farrington, 2006). These constructs therefore do not represent interchangeable predictors of delinquent behavior. Rather, they mark distinct but theoretically connected domains: dispositional risk, moral-cognitive justification, and social-cognitive constraint. What remains less directly examined is how these domains can be modeled together within a single theory-informed account of delinquent behavior among justice-involved adolescents. This issue is particularly relevant in culturally and institutionally specific contexts, including Saudi social observation centers, where justice-involved adolescents are studied within a legal, cultural, and institutional framework that may differ from community or non-institutional research settings.

**FIGURE 1:**
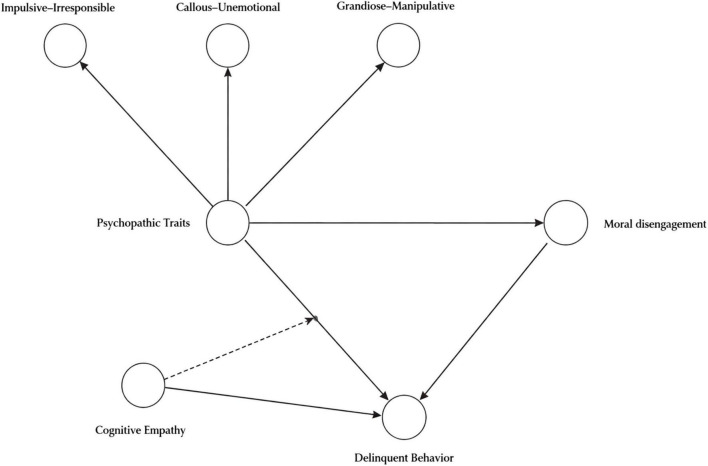
Proposed theory-informed structural model of youth psychopathic traits, moral disengagement, cognitive empathy, and delinquent behavior. The figure presents the hypothesized concurrent structural associations. The indirect path through moral disengagement and the moderating role of cognitive empathy were specified as theory-informed statistical associations rather than temporal or causal mechanisms.

Building on this framing, the present study tested a theory-informed cross-sectional structural model of delinquent behavior among justice-involved adolescents. The model examined whether youth psychopathic traits were directly associated with delinquent behavior, whether this association included a statistically estimated indirect component via moral disengagement, and whether cognitive empathy qualified the direct association between youth psychopathic traits and delinquent behavior. Because the data were cross-sectional, the indirect path through moral disengagement was interpreted as a statistically estimated concurrent indirect association rather than evidence of temporal mediation or a causal mechanism (Cole and Maxwell, 2003; Maxwell and Cole, 2007). The interaction involving cognitive empathy was likewise interpreted as a conditional concurrent association rather than evidence of a causal buffering effect. The proposed structural model is presented in Figure 1.

On this basis, the preceding literature supports a set of directional expectations for the study hypotheses. Youth psychopathic traits were expected to be positively associated with delinquent behavior, consistent with meta-analytic evidence linking juvenile psychopathic traits to delinquency and recidivism-related outcomes (Asscher et al., 2011; Geerlings et al., 2020). These traits were also expected to be positively associated with moral disengagement, given prior evidence connecting psychopathy-related or callous–unemotional traits with moral disengagement, while recognizing that this evidence includes complex, reciprocal, and correlational patterns rather than a confirmed unidirectional pathway (Muratori et al., 2017; Paciello et al., 2020; Gomez Tabares et al., 2025). Moral disengagement was expected to be positively associated with delinquent behavior because it reflects moral-cognitive mechanisms through which harmful conduct may be justified and self-sanctions weakened, and because prior adolescent and youth-focused evidence links moral disengagement to antisocial, aggressive, violent, and transgressive behavior (Bandura et al., 1996; Bandura, 2002; Hyde et al., 2010; Paciello et al., 2008; Shulman et al., 2011; Luo and Bussey, 2023). Cognitive empathy was expected to be negatively associated with delinquent behavior, consistent with meta-analytic evidence linking lower cognitive empathy to offending (Jolliffe and Farrington, 2004; van Langen et al., 2014). The moderation hypothesis was specified as theory-informed and exploratory in direction because direct evidence for cognitive empathy as a moderator of the psychopathic traits–delinquency association remains limited.

Accordingly, the study tested the following hypotheses:

*H1*: Youth psychopathic traits will be positively associated with delinquent behavior.

*H2*: Youth psychopathic traits will be positively associated with moral disengagement.

*H3*: Moral disengagement will be positively associated with delinquent behavior.

*H4*: Youth psychopathic traits will show a positive indirect statistical association with delinquent behavior via moral disengagement.

*H5*: Cognitive empathy will be negatively associated with delinquent behavior.

*H6*: Cognitive empathy will moderate the concurrent direct association between youth psychopathic traits and delinquent behavior, such that this association is expected to be weaker at higher levels of cognitive empathy. Given the limited direct evidence for this specific moderating role, this hypothesis was treated as theory-informed and exploratory in direction.

Consistent with the cross-sectional design, these hypotheses concern concurrent structural associations rather than temporal or causal effects.

## 2 Materials and methods

### 2.1 Research design and setting

This study used a cross-sectional correlational design based on self-report questionnaire data collected from adolescents residing in social observation centers in Saudi Arabia. The study was designed to examine theory-informed concurrent associations among youth psychopathic traits, moral disengagement, cognitive empathy, and delinquent behavior. Because the data were collected at a single time point, the estimated direct, indirect, and interaction paths were interpreted as concurrent structural associations rather than evidence of temporal ordering or causal effects.

### 2.2 Participants

The study included 171 juvenile offenders residing in social observation centers across Saudi Arabia. Participants had a mean age of 16.06 years (SD = 1.10) and reported an average of 1.89 admissions to social observation centers. Data were collected from nine social observation centers across major regions of Saudi Arabia, providing geographic coverage beyond a single institutional site. Table 1 summarizes the sample’s nationality, educational level, and parental-status distribution.

**TABLE 1:** Participant characteristics (*N* = 171).

Variable	*n*	%
Age (years), M (SD)	16.06 (1.10)	–
Reported number of admissions to social observation centers, M (SD) [Range]	1.89 (0.88) [1–4]	
Nationality		
Saudi	133	77.8
Non-Saudi	38	22.2
Educational level		
Secondary school	102	59.6
Middle school	37	21.6
Elementary school	32	18.7
Parental status		
Both parents alive	134	78.4
Father deceased	31	18.1
Mother deceased	4	2.3
Both parents deceased	2	1.2

### 2.3 Sampling technique and recruitment procedure

Participants were recruited through coordination with administrative and social work staff at social observation centers across Saudi Arabia. Recruitment followed a consecutive convenience sampling approach, whereby juveniles who met the predefined eligibility criteria and were present at the participating centers during the data-collection period were invited to take part. This approach was selected because the target population consisted of justice-involved adolescents residing in institutional settings, where access to participants is governed by administrative approval, institutional availability, eligibility screening, and ethical safeguards. Accordingly, probability sampling from the broader population of justice-involved adolescents was not practicable in this context.

This sampling strategy was appropriate for the study objective, which was to examine delinquency-related psychological constructs among justice-involved adolescents currently residing in Saudi social observation centers. Consecutive recruitment also helped reduce researcher discretion in participant selection by inviting eligible residents who were available during the data-collection period. Therefore, the sample should be interpreted as a sample of eligible participating juveniles recruited from social observation centers, rather than as a probability-based representative sample of all justice-involved adolescents in Saudi Arabia.

### 2.4 Inclusion and exclusion criteria

Eligibility criteria were as follows: (a) being officially classified as a juvenile offender residing in a social observation center at the time of data collection, (b) being between 13 and 18 years of age, and (c) having sufficient Arabic-language proficiency to comprehend and respond to the questionnaire items. Eligibility was based on language proficiency rather than nationality. Accordingly, non-Arab youth were not excluded on the basis of nationality alone; they were excluded only if they were unable to understand the Arabic instructions and questionnaire items sufficiently to complete the Arabic-language measures reliably. This criterion was used to minimize comprehension-related measurement error and to support the validity of self-report responses.

### 2.5 Data collection procedure

Questionnaires were administered individually in a supervised setting during scheduled data-collection sessions at each center. Standardized instructions were used to support consistent administration across sites. Trained social workers assisted with logistical arrangements and were available to clarify procedural questions; however, they were instructed not to explain, interpret, or suggest responses to substantive questionnaire items. Administrative and social work staff facilitated access to eligible available participants but were instructed not to nominate participants selectively on the basis of behavioral, clinical, or institutional judgments. Participation was voluntary, and no incentives were provided.

### 2.6 Ethics approval and consent to participate

All procedures involving human participants were conducted in accordance with the ethical standards of the institutional Research Ethics Committee and with the 1964 Declaration of Helsinki and its later amendments or comparable ethical standards. The study protocol was reviewed and approved by the Research Ethics Committee at the Islamic University of Madinah (approval no. 5/2025; September 17, 2025).

Because participants were minors residing in social observation centers, participation procedures followed the consent and assent process approved by the Research Ethics Committee. Institutional permission was obtained in accordance with applicable center procedures, and adolescents provided assent before participation. Participation was voluntary and had no effect on participants’ legal status, institutional decisions, or access to services. The research team did not collect names, identification numbers, or other direct identifiers. The analytic dataset was therefore de-identified, and all study materials were stored securely and used for research purposes only.

### 2.7 Instruments

All instruments were administered in Arabic. For the purposes of the present study, Arabic item content was prepared before data collection through a structured translation and review process. The original items were forward translated into Arabic and then reviewed by bilingual specialists for semantic consistency, linguistic clarity, age-appropriate wording, and suitability for administration among justice-involved adolescents in social observation centers. Wording discrepancies were reconciled before the final Arabic item set was prepared. The draft Arabic items were also pilot checked to ensure that the item wording, instructions, and response format were comprehensible and did not create ambiguity in the study setting.

After data collection, the psychometric performance of the Arabic item set in the present sample was evaluated through the PLS-SEM measurement model, including standardized outer loadings, internal consistency reliability, composite reliability, AVE, and discriminant validity.

#### 2.7.1 Youth psychopathic traits

Youth psychopathic traits were assessed using the Youth Psychopathic Traits Inventory–Short Version (YPI-S; van Baardewijk et al., 2010; Colins et al., 2012). The YPI-S includes 18 items designed to assess psychopathy-related traits in adolescents across three dimensions: grandiose–manipulative/interpersonal, callous–unemotional/affective, and impulsive–irresponsible/behavioral traits. Responses are rated on a four-point scale ranging from 0 (does not apply at all) to 3 (applies very well), with higher scores indicating higher levels of youth psychopathic traits.

The initial YPI-S development study reported Cronbach’s alpha values ranging from 0.69 to 0.71 for the dimension scores. In a direct administration of the 18-item YPI-S, Colins et al. (2012) reported α = 0.78 for the total score, α = 0.76 for the grandiose-manipulative/interpersonal dimension, α = 0.66 for the callous-unemotional/affective dimension, and α = 0.66 for the impulsive-irresponsible/behavioral dimension.

#### 2.7.2 Moral disengagement

Moral disengagement was assessed using the eight-item Propensity to Morally Disengage Scale developed by Moore et al. (2012), which was grounded in Bandura et al.’s (1996) moral disengagement framework. The scale includes one item representing each of Bandura’s eight moral disengagement mechanisms: moral justification, euphemistic labeling, advantageous comparison, displacement of responsibility, diffusion of responsibility, distortion of consequences, dehumanization, and attribution of blame. In the present study, the items were administered using a five-point Likert scale ranging from 1 (strongly disagree) to 5 (strongly agree), with higher scores indicating a greater propensity to morally disengage.

In the original validation study, the eight-item version demonstrated acceptable internal consistency (α = 0.80) and supported a one-factor structure, χ^2^(20) = 27, RMSEA = 0.045, SRMR = 0.043, NNFI = 0.98, CFI = 0.99. Across subsequent samples reported by Moore et al. (2012), internal consistency for the eight-item version ranged from α = 0.70 to 0.90.

#### 2.7.3 Cognitive empathy

Cognitive empathy was measured using the cognitive-empathy subscale of the Basic Empathy Scale (BES; Jolliffe and Farrington, 2004). The BES was developed from an initial 40-item pool and finalized as a 20-item self-report measure based on a two-dimensional formulation of empathy, distinguishing cognitive empathy from affective empathy. In the original validation study, the final scale included nine cognitive-empathy items and eleven affective-empathy items. Consistent with the theoretical focus of the present study, the cognitive-empathy subscale was used to assess adolescents’ ability to understand others’ emotional states. Items were rated on a five-point Likert scale ranging from 1 (strongly disagree) to 5 (strongly agree), with higher scores indicating greater cognitive empathy.

In Jolliffe and Farrington’s original validation study, the cognitive-empathy subscale showed acceptable internal consistency (α = 0.79), whereas the affective-empathy subscale showed higher internal consistency (α = 0.85). Confirmatory factor analysis supported the two-factor structure of the BES, with acceptable model fit indices (GFI = 0.89, AGFI = 0.86, RMS = 0.06).

#### 2.7.4 Delinquent behavior

Delinquent behavior was assessed using a 10-item self-report delinquency measure adapted from prior self-report delinquency measures based on the work of Elliott and colleagues (Elliott et al., 1985, 1989). The items assessed adolescents’ involvement in different delinquent acts during the past 12 months, including theft, property damage, physical assault, weapon carrying, and use of force to obtain money or property. Participants reported how often they had engaged in each behavior using a five-point frequency scale: 0 (never), 1 (once), 2 (two or three times), 3 (four or five times), and 4 (six or more times). Item scores were summed to produce a total delinquency index ranging from 0 to 40, with higher scores indicating greater self-reported delinquent involvement.

Xiong and Huang (2011) reported good internal consistency for a 10-item self-report delinquency measure assessing past-year delinquent behavior (α = 0.83). Because participants in the present study were residing in social observation centers at the time of data collection, this retrospective 12-month measure did not distinguish whether reported behaviors occurred before admission, during institutional residence, or across both contexts.

### 2.8 Data analysis methods

Before model estimation, the dataset was screened for item-level missing responses. No missing responses were identified across the study variables, as questionnaires were completed in full during supervised administration. Accordingly, the analytic sample remained unchanged (*N* = 171) throughout the measurement and structural model analyses.

The analytical strategy was selected to match the study objectives, the hypothesized model structure, and the measurement characteristics of the data. The study examined associations among multi-item self-report constructs, including direct structural paths, a statistically estimated indirect component through moral disengagement, and an interaction involving cognitive empathy. Partial least-squares structural equation modeling (PLS-SEM) was therefore used to estimate the theory-informed structural model in SmartPLS 4. PLS-SEM was appropriate because it allows simultaneous assessment of reflective measurement models and structural relations among latent constructs, with an emphasis on explained variance and prediction-oriented structural associations (Hair et al., 2019, 2022). The use of PLS-SEM was also consistent with the structure of the data, as the study variables were assessed using multi-item Likert-type or frequency-response scales and modeled as latent constructs rather than as single observed indicators.

Although PLS-SEM does not require the same distributional assumptions as covariance-based SEM, the analysis incorporated relevant diagnostic checks to evaluate the appropriateness and robustness of the analytical strategy. These checks included evaluation of indicator reliability, internal consistency reliability, convergent validity, and discriminant validity; assessment of collinearity using VIF values; nonparametric bootstrapping for direct, indirect, and interaction effects; predictive assessment; and an a priori power analysis for sample-size adequacy. The analysis followed the recommended two-step approach, in which the measurement model was evaluated before assessing the structural model (Hair et al., 2019, 2022). Given the cross-sectional design, all direct, indirect, and interaction paths were interpreted as concurrent structural associations rather than evidence of temporal ordering or causal effects.

For the reflective measurement models, internal consistency reliability was assessed using Cronbach’s alpha, composite reliability, and rho_A. Convergent validity was examined using standardized indicator loadings and the average variance extracted (AVE). Discriminant validity was evaluated using the heterotrait–monotrait ratio of correlations (HTMT) and the Fornell–Larcker criterion, where applicable. Decisions regarding item retention were guided by both statistical criteria and theoretical considerations.

After satisfactory measurement model evaluation, the structural model was assessed by examining collinearity among predictor constructs using variance inflation factors (VIFs), estimating path coefficients, and evaluating explained variance (*R*^2^). Effect sizes (f^2^) were calculated to assess the relative contribution of each predictor. The significance of the direct paths, the statistically estimated indirect component via moral disengagement, and the interaction effect involving cognitive empathy were tested using nonparametric bootstrapping with 5,000 resamples, consistent with recommended PLS-SEM practice (Hair et al., 2022).

The interaction effect was estimated in SmartPLS 4 using the two-stage approach. In the first stage, latent variable scores were obtained for youth psychopathic traits and cognitive empathy. In the second stage, an interaction term was created as the product of these latent scores and specified as predicting delinquent behavior together with the main-effect predictors. The significance of the interaction effect was assessed using the same bootstrapping procedure.

Because the study adopted a prediction-oriented PLS-SEM approach, predictive relevance was also assessed using PLSpredict, following recommended procedures for predictive assessment in PLS-SEM (Shmueli et al., 2019; Hair et al., 2022). The assessment focused on Q^2^_predict, RMSE, and MAE for the substantive endogenous target constructs. The PLSpredict results were interpreted conservatively as supplementary evidence of predictive relevance for the endogenous constructs examined.

Sample size adequacy was evaluated using an a priori power analysis rather than heuristic rules such as the 10-times rule, consistent with recommended practice in PLS-SEM research (Hair et al., 2019; Kock and Hadaya, 2018). The most complex structural equation included four predictors: youth psychopathic traits, moral disengagement, cognitive empathy, and the interaction term. A regression-based power analysis assuming a medium effect size (*f*^2^ = 0.15), α = 0.05, and power of 0.95 indicated a minimum required sample size of 129. The achieved sample size (*N* = 171) exceeded this threshold, providing adequate power for detecting effects of at least moderate size in the hypothesized structural relations. Smaller interaction effects, however, should be interpreted cautiously.

### 2.9 Assessment of common method variance

Because all focal variables were assessed using self-report questionnaires administered at a single time point, common method variance (CMV) was considered a plausible source of bias. Procedural safeguards were used during data collection, including voluntary participation, de-identification of responses for analysis, emphasis on confidentiality, instructions to respond honestly, and the use of established instruments with clear wording and varied response formats. These procedures were intended to reduce some method-related response tendencies, but they cannot eliminate CMV risk in a cross-sectional self-report design (Podsakoff et al., 2003).

Statistical diagnostics were conducted as descriptive sensitivity checks rather than as evidence that common method bias was absent, particularly because Harman’s single-factor test is not considered sufficient for ruling out common method bias. Harman’s single-factor test indicated that the first unrotated factor accounted for 27.31% of the total variance. In addition, a common latent factor (CLF) sensitivity analysis was implemented in SmartPLS 4. The key structural path coefficients did not change in direction or statistical inference after adding the method factor, with relative changes ranging from 0.9 to 14.5%. These findings suggest that the main structural results were not materially altered in these sensitivity checks. However, because the study used a single-source, cross-sectional self-report design, these diagnostics cannot rule out CMV, which was therefore treated as a methodological limitation. A summary of these diagnostic checks is presented in Table 2.

**TABLE 2:** Common method variance assessment: summary of diagnostic checks.

Diagnostic approach	Result	Interpretation
Harman’s single-factor test	First factor accounted for 27.31% of total variance	No single dominant factor accounted for the majority of item covariance; treated as an initial diagnostic check
Common latent factor (CLF) analysis		
YPT → DEL	Δβ = 7.7%	Small relative change; direction and statistical inference unchanged
CE → DEL	Δβ = 0.9%	Minimal relative change; direction and statistical inference unchanged
CE × YPT → DEL	Δβ = 3.3%	Minimal relative change; direction and statistical inference unchanged
MD → DEL	Δβ = 14.5%	Modest relative change; direction and statistical inference unchanged
Alternative CLF specification	Path coefficients were virtually identical	Results were robust to the alternative CLF specification
Overall conclusion	Path estimates were stable in direction and statistical inference across diagnostics	CMV diagnostics did not materially alter the substantive inferences

YPT, Youth psychopathic traits; MD, moral disengagement; CE, cognitive empathy; DEL, self-reported delinquent behavior; CLF, common latent factor; CMV, common method variance. Δβ refers to the relative change in standardized path coefficients after adding the common latent factor.

## 3 Results

### 3.1 Measurement model

#### 3.1.1 Internal consistency reliability

Internal consistency reliability was evaluated using Cronbach’s alpha, rho_A, and composite reliability (rho_C). All constructs exceeded the recommended 0.70 threshold, indicating adequate internal consistency reliability (Hair et al., 2022). The three lower-order dimensions of youth psychopathic traits showed adequate to strong reliability: grandiose–manipulative traits (α = 0.894, rho_A = 0.897, rho_C = 0.919), callous–unemotional traits (α = 0.879, rho_A = 0.880, rho_C = 0.909), and impulsive–irresponsible traits (α = 0.874, rho_A = 0.877, rho_C = 0.905). The overall psychopathic traits construct also showed high internal consistency (α = 0.941, rho_A = 0.942, rho_C = 0.947). Because this construct represented a multidimensional psychopathy-related trait specification rather than a simple unidimensional scale, the high reliability estimate was interpreted cautiously and was not treated as evidence of unidimensionality. Moral disengagement (α = 0.840, rho_A = 0.865, rho_C = 0.876), cognitive empathy (α = 0.881, rho_A = 0.898, rho_C = 0.902), and delinquent behavior (α = 0.878, rho_A = 0.887, rho_C = 0.903) also demonstrated adequate reliability. Table 3 summarizes the reliability and convergent-validity indices.

**TABLE 3:** Construct reliability and convergent validity.

Construct	Cronbach’s α	rho_A	rho_C	AVE
Callous–unemotional	0.879	0.880	0.909	0.624
Cognitive empathy	0.881	0.898	0.902	0.508
Delinquent behavior	0.878	0.887	0.903	0.510
Grandiose–manipulative	0.894	0.897	0.919	0.656
Impulsive–irresponsible	0.874	0.877	0.905	0.614
Moral disengagement	0.840	0.865	0.876	0.505
Psychopathic traits	0.941	0.942	0.947	0.501

α, Cronbach’s alpha; rho_A, Dijkstra–Henseler’s reliability coefficient; rho_C, composite reliability; AVE, average variance extracted.

#### 3.1.2 Convergent validity

Convergent validity was evaluated using standardized outer loadings and average variance extracted (AVE). All constructs met the recommended AVE threshold of 0.50. AVE values were 0.656 for grandiose–manipulative traits, 0.624 for callous–unemotional traits, 0.614 for impulsive–irresponsible traits, 0.510 for delinquent behavior, 0.508 for cognitive empathy, 0.505 for moral disengagement, and 0.501 for the overall psychopathic traits construct. These results indicated that all constructs met the minimum recommended AVE criterion, although several AVE values were close to the threshold and were therefore interpreted conservatively.

Most standardized outer loadings were close to or above the recommended 0.70 level. Several indicators loaded below 0.70 but remained above 0.50; these indicators were retained because their corresponding constructs met the AVE and composite reliability criteria, and further deletion was not required. Item N3 from the moral disengagement scale was not retained in the final measurement model because its removal allowed the AVE of the moral disengagement construct to meet the recommended threshold for convergent validity. This decision was made at the indicator level and was retained only because the remaining moral disengagement indicators continued to provide acceptable internal consistency reliability, convergent validity, and adequate construct coverage for the present structural model. The retained indicators S6 and S16 loaded on their respective lower-order dimensions, grandiose–manipulative traits and impulsive–irresponsible traits, and contributed to the overall psychopathic traits construct. Overall, the measurement model results supported adequate internal consistency reliability and convergent validity.

#### 3.1.3 Discriminant validity

Discriminant validity was evaluated primarily using the heterotrait–monotrait ratio of correlations (HTMT), with the Fornell–Larcker criterion reported as supplementary evidence. Because psychopathic traits were represented by an overall psychopathy-related construct together with its three lower-order dimensions, discriminant validity was not interpreted between the overall construct and its constituent dimensions. These associations were expected to be high because the lower-order dimensions formed the conceptual and measurement basis of the broader psychopathic traits construct.

As shown in Table 4, the HTMT values for the substantively distinct constructs included in the structural model were below the conservative 0.85 threshold. Specifically, HTMT values were 0.319 for psychopathic traits and moral disengagement, 0.417 for psychopathic traits and cognitive empathy, 0.424 for psychopathic traits and delinquent behavior, 0.227 for moral disengagement and cognitive empathy, 0.349 for moral disengagement and delinquent behavior, and 0.526 for cognitive empathy and delinquent behavior. The high HTMT values between the overall psychopathic traits construct and its lower-order dimensions were not interpreted as evidence against discriminant validity because these dimensions were specified as components of the broader construct rather than as independent constructs. These results supported discriminant validity among the substantively distinct constructs used to estimate the structural paths.

**TABLE 4:** Heterotrait–monotrait ratio of correlations (HTMT).

Construct	1	2	3	4	5	6	7
Callous–unemotional	—	–	–	–	–	–	—
Cognitive empathy	0.309						
Delinquent behavior	0.376	0.526					
Grandiose–manipulative	0.771	0.436	0.460				
Impulsive–irresponsible	0.825	0.404	0.329	0.744			
Moral disengagement	0.290	0.227	0.349	0.266	0.324		
Psychopathic traits	0.990	0.417	0.424	0.959	0.981	0.319	

HTMT, heterotrait–monotrait ratio of correlations.

The Fornell–Larcker results, reported in Table 5, provided supplementary support for discriminant validity among the substantively distinct constructs. The square roots of AVE were 0.708 for psychopathic traits, 0.711 for moral disengagement, 0.713 for cognitive empathy, and 0.714 for delinquent behavior. For these structural-model constructs, each square root of AVE exceeded its correlations with the other substantively distinct constructs. However, the Fornell–Larcker criterion was not used to infer discriminant validity between the overall psychopathic traits construct and its own lower-order dimensions. Overall, the results supported adequate discriminant validity for the substantively distinct constructs included in the structural model.

**TABLE 5:** Fornell–Larcker criterion.

Construct	1	2	3	4	5	6	7
Callous–unemotional	0.790	0.713	0.714	0.810	0.784	0.711	0.708
Cognitive empathy	−0.289						
Delinquent behavior	0.332	−0.502					
Grandiose–manipulative	0.687	−0.390	0.413				
Impulsive–irresponsible	0.729	−0.370	0.294	0.662			
Moral disengagement	0.270	−0.181	0.318	0.248	0.290		
Psychopathic traits	0.903	−0.394	0.391	0.883	0.890	0.302	

Diagonal values represent the square root of AVE; off-diagonal values represent inter-construct correlations.

### 3.2 Structural model evaluation

Following the satisfactory assessment of the measurement model, the structural model was evaluated following the recommended PLS-SEM assessment sequence described by Hair et al. (2022). The evaluation included collinearity diagnostics, assessment of structural path coefficients, explained variance (*R*^2^), effect sizes (*f*^2^), the specific indirect effect, the interaction effect, and predictive relevance using PLSpredict. Bootstrapped significance testing was conducted using 5,000 bootstrap subsamples.

#### 3.2.1 Collinearity assessment

Collinearity was assessed using variance inflation factor (VIF) values for the predictor constructs in the structural model. The VIF values were 1.000 for psychopathic traits predicting moral disengagement, 1.316 for psychopathic traits predicting delinquent behavior, 1.154 for moral disengagement predicting delinquent behavior, 1.275 for cognitive empathy predicting delinquent behavior, and 1.125 for the interaction term predicting delinquent behavior. For the lower-order dimensions of psychopathic traits, the VIF values were 1.000 for psychopathic traits predicting callous–unemotional traits, 1.000 for psychopathic traits predicting grandiose–manipulative traits, and 1.000 for psychopathic traits predicting impulsive–irresponsible traits. All VIF values were below the conservative threshold of 3.3, indicating no substantial collinearity concerns and supporting the stability of the structural path estimates.

#### 3.2.2 Path coefficients and significance tests

As shown in Table 6, psychopathic traits were positively associated with moral disengagement [β = 0.302, *t* = 4.234, *p* < 0.001, 95% CI (0.169, 0.449)] and delinquent behavior [β = 0.217, *t* = 3.588, *p* < 0.001, 95% CI (0.089, 0.326)]. Moral disengagement was also positively associated with delinquent behavior [β = 0.153, *t* = 2.734, *p* = 0.006, 95% CI (0.047, 0.268)]. Cognitive empathy showed a negative association with delinquent behavior [β = −0.344, *t* = 4.639, *p* < 0.001, 95% CI (−0.497, −0.210)].

**TABLE 6:** Structural path coefficients.

Path	β	*t*	*p*	95% CI
Youth psychopathic traits → Moral disengagement	0.302	4.234	<0.001	[0.169, 0.449]
Youth psychopathic traits → Delinquent behavior	0.217	3.588	<0.001	[0.089, 0.326]
Moral disengagement → Delinquent behavior	0.153	2.734	0.006	[0.047, 0.268]
Cognitive empathy → Delinquent behavior	−0.344	4.639	<0.001	[−0.497, −0.210]
Cognitive empathy × Youth psychopathic traits → Delinquent behavior	−0.175	2.676	0.007	[−0.311, −0.050]

β, standardized path coefficient; CI, bootstrap confidence interval.

The interaction term between cognitive empathy and psychopathic traits was statistically significant [β = −0.175, *t* = 2.676, *p* = 0.007, 95% CI (−0.311, −0.050)]. This finding indicates that cognitive empathy qualified the concurrent association between psychopathic traits and delinquent behavior. The negative coefficient was consistent with a weaker positive association between psychopathic traits and delinquent behavior at higher levels of cognitive empathy. This interaction was interpreted as a concurrent conditional association rather than evidence of a causal buffering effect.

#### 3.2.3 Effect size (*f*^2^)

As shown in Table 7, effect size estimates (*f*^2^) were examined to assess the relative contribution of each predictor to the explained variance of the endogenous constructs. For moral disengagement, psychopathic traits showed a small effect (*f*^2^ = 0.101). For delinquent behavior, cognitive empathy produced the largest relative *f*^2^ contribution among the predictors (*f*^2^ = 0.146), which remained slightly below the conventional medium-effect threshold of 0.15. Psychopathic traits showed a small effect on delinquent behavior (*f*^2^ = 0.056), whereas moral disengagement showed a smaller effect (*f*^2^ = 0.032). The interaction term between cognitive empathy and psychopathic traits also showed a small effect on delinquent behavior (*f*^2^ = 0.053). Overall, the *f*^2^ results indicated that cognitive empathy contributed most strongly to the explained variance in delinquent behavior, followed by psychopathic traits, the interaction term, and moral disengagement.

**TABLE 7:** Effect sizes (*f*^2^).

Path	*f* ^2^	Interpretation
Youth psychopathic traits → Moral disengagement	0.101	Small
Youth psychopathic traits → Delinquent behavior	0.056	Small
Moral disengagement → Delinquent behavior	0.032	Small
Cognitive empathy → Delinquent behavior	0.146	Small, approaching medium
Cognitive empathy × Youth psychopathic traits → Delinquent behavior	0.053	Small

*f*^2^ = effect size. Interpretations follow the conventional thresholds commonly used in PLS-SEM:0.02 = small, 0.15 = medium, and 0.35 = large.

#### 3.2.4 Explained variance (*R*^2^), specific indirect effect, and interaction effect

**FIGURE 2:**
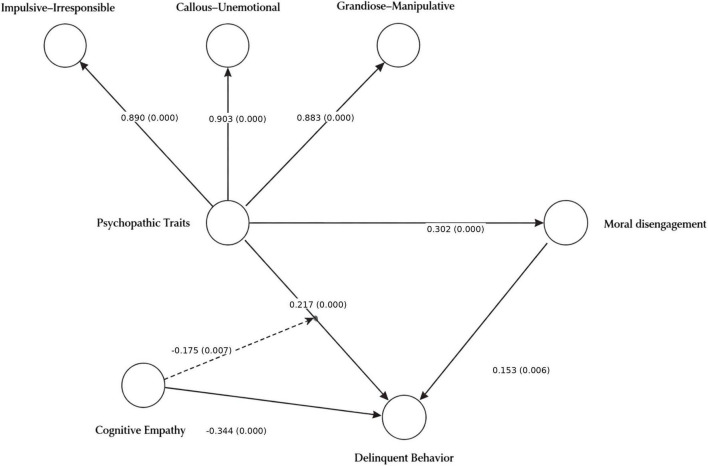
Structural model of psychopathic traits, moral disengagement, cognitive empathy, and delinquent behavior. Standardized path coefficients are shown. Values inside endogenous constructs and lower-order psychopathic-trait dimensions represent *R*^2^ values from the estimated model. Paths to the three psychopathic-trait dimensions represent the hierarchical measurement specification, not substantive structural hypotheses.

As shown in Table 8, the model explained 36.4% of the variance in delinquent behavior (*R*^2^ = 0.364, adjusted *R*^2^ = 0.348). Youth psychopathic traits explained 9.1% of the variance in moral disengagement (*R*^2^ = 0.091, adjusted *R*^2^ = 0.086). These results indicate that the model accounted for a larger proportion of variance in delinquent behavior than in moral disengagement. The structural model is summarized in Figure 2.

**TABLE 8:** Explained variance (*R*^2^).

Endogenous construct	*R* ^2^	Adjusted *R*^2^	95% CI for *R*^2^
Moral disengagement	0.091	0.086	[0.028, 0.201]
Delinquent behavior	0.364	0.348	[0.263, 0.542]

*R*^2^, coefficient of determination; CI, bootstrap confidence interval.

**FIGURE 3:**
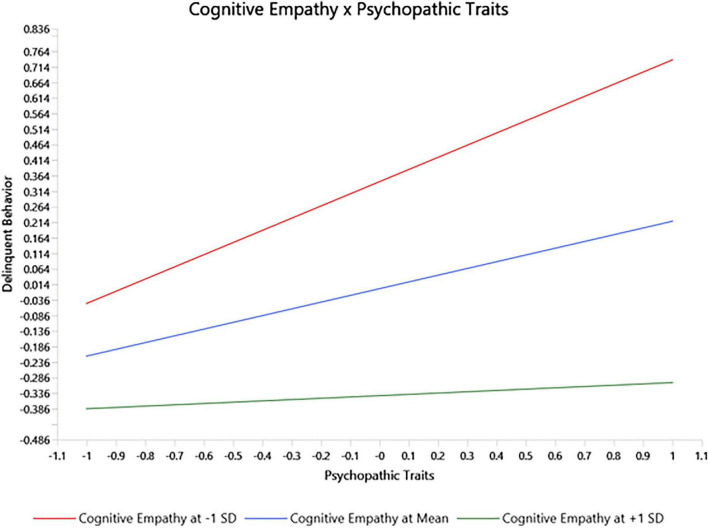
Interaction between psychopathic traits and cognitive empathy in predicting delinquent behavior. Simple slopes are plotted at low (−1 SD), mean, and high (+1 SD) levels of cognitive empathy.

As shown in Table 9, the specific indirect effect from youth psychopathic traits to delinquent behavior via moral disengagement was statistically significant [β = 0.046, *t* = 2.174, *p* = 0.030, 95% CI (0.014, 0.096)]. This result indicates that moral disengagement accounted for a small but statistically detectable indirect component of the concurrent association between youth psychopathic traits and delinquent behavior. The interaction between cognitive empathy and youth psychopathic traits was reported above in Table 6 and is examined further in the simple-slope plot in Figure 3.

**TABLE 9:** Specific indirect effect.

Indirect path	β	*t*	*p*	95% CI
Youth psychopathic traits → Moral disengagement → Delinquent behavior	0.046	2.174	0.030	[0.014, 0.096]

β, standardized indirect effect; CI, bootstrap confidence interval.

#### 3.2.5 Supplementary sensitivity analysis with age and admissions as covariates

A supplementary sensitivity analysis was conducted to examine whether the main structural findings remained stable after accounting for age and reported number of admissions to social observation centers. Age and admissions were added as covariates predicting moral disengagement and delinquent behavior. Neither age nor admissions was significantly associated with moral disengagement or delinquent behavior: age did not predict moral disengagement (β = −0.090, *p* = 0.268) or delinquent behavior (β = 0.044, *p* = 0.402), and admissions did not predict moral disengagement (β = 0.033, *p* = 0.721) or delinquent behavior (β = −0.043, *p* = 0.457). The substantive pattern of findings remained stable: youth psychopathic traits continued to predict moral disengagement (β = 0.330, *p* < 0.001) and delinquent behavior (β = 0.196, *p* = 0.005), moral disengagement continued to predict delinquent behavior (β = 0.152, *p* = 0.007), cognitive empathy remained negatively associated with delinquent behavior (β = −0.347, *p* < 0.001), and the interaction between cognitive empathy and youth psychopathic traits remained significant (β = −0.174, *p* = 0.007). The specific indirect effect through moral disengagement also remained significant (β = 0.050, *p* = 0.045). Overall, the main findings were not materially altered after accounting for these background variables.

#### 3.2.6 Supplementary exploratory dimension-level analysis

A supplementary exploratory dimension-level analysis was conducted to examine whether the associations observed for the overall youth psychopathic traits construct were broadly shared across its lower-order dimensions or were more evident for specific dimensions. In this secondary analysis, the overall psychopathic traits construct was not included as a predictor; instead, grandiose–manipulative, callous–unemotional, and impulsive–irresponsible traits were modeled simultaneously as predictors of moral disengagement and delinquent behavior. Cognitive empathy and moral disengagement were retained as predictors of delinquent behavior.

Inner VIF values ranged from 1.126 to 2.564, indicating no substantial collinearity concerns among the predictors in the exploratory dimension-level model. When the three dimensions were entered simultaneously, only grandiose–manipulative traits showed a statistically significant unique direct association with delinquent behavior [β = 0.225, 95% CI (0.033, 0.388), *p* = 0.014]. Callous–unemotional traits [β = 0.111, 95% CI (−0.064, 0.302), *p* = 0.231] and impulsive–irresponsible traits [β = −0.107, 95% CI (−0.329, 0.119), *p* = 0.359] were not significantly associated with delinquent behavior. None of the three dimensions significantly predicted moral disengagement: grandiose–manipulative traits [β = 0.042, 95% CI (−0.193, 0.267), *p* = 0.717], callous–unemotional traits [β = 0.065, 95% CI (−0.226, 0.360), *p* = 0.663], and impulsive–irresponsible traits [β = 0.241, 95% CI (−0.016, 0.518), *p* = 0.078]. Moral disengagement was positively associated with delinquent behavior [β = 0.187, 95% CI (0.075, 0.302), *p* = 0.001], and cognitive empathy was negatively associated with delinquent behavior [β = −0.372, 95% CI (−0.529, −0.228), *p* < 0.001].

The dimension-specific indirect effects through moral disengagement did not reach statistical significance. The bootstrap confidence intervals included zero for impulsive–irresponsible traits [β = 0.045, 95% CI (−0.003, 0.128), *p* = 0.192], callous–unemotional traits [β = 0.012, 95% CI (−0.051, 0.067), *p* = 0.678], and grandiose–manipulative traits [β = 0.008, 95% CI (−0.038, 0.055), *p* = 0.734]. Overall, this supplementary exploratory analysis indicated that, when the three lower-order dimensions were modeled simultaneously, only grandiose–manipulative traits showed a statistically significant unique direct association with delinquent behavior. No dimension-specific indirect effect through moral disengagement reached statistical significance. These findings should be interpreted cautiously because the analysis was exploratory, involved simultaneous modeling of conceptually related dimensions, and was not specified as the primary test of the study hypotheses.

#### 3.2.7 Simple slopes and interaction plot

To clarify the statistically significant interaction between youth psychopathic traits and cognitive empathy, the interaction was plotted at low (−1 SD), mean, and high (+1 SD) levels of cognitive empathy. As shown in Figure 3, the association between youth psychopathic traits and delinquent behavior was positive and strongest when cognitive empathy was low. The slope remained positive but was weaker at the mean level of cognitive empathy, and it became much weaker at high levels of cognitive empathy. This pattern is consistent with the negative interaction coefficient reported in the structural model and indicates that the positive concurrent association between youth psychopathic traits and delinquent behavior was weaker at higher levels of cognitive empathy. Because the study used a cross-sectional design, this pattern was interpreted as a conditional concurrent association rather than evidence that cognitive empathy causally buffered the effect of youth psychopathic traits.

#### 3.2.8 Hypothesis testing summary

Overall, the structural results were consistent with the hypothesized directions. Youth psychopathic traits were positively associated with delinquent behavior, consistent with H1, and were positively associated with moral disengagement, consistent with H2. Moral disengagement was positively associated with delinquent behavior, consistent with H3. The statistically estimated indirect effect from youth psychopathic traits to delinquent behavior through moral disengagement was statistically significant, consistent with H4; however, this effect was interpreted as a small concurrent indirect association rather than evidence of temporal or causal mediation. Cognitive empathy was negatively associated with delinquent behavior, consistent with H5. Finally, the significant negative interaction between cognitive empathy and youth psychopathic traits, together with the simple-slope pattern, was consistent with H6, indicating that the positive concurrent association between youth psychopathic traits and delinquent behavior was weaker at higher levels of cognitive empathy.

#### 3.2.9 Assessment of prediction using PLSpredict

As shown in Table 10, predictive relevance was assessed using PLSpredict for the substantive endogenous target constructs in the structural model. Moral disengagement showed positive but limited predictive relevance (Q^2^_predict = 0.074, RMSE = 0.970, MAE = 0.789). Delinquent behavior showed a higher positive Q^2^_predict value (Q^2^_predict = 0.281, RMSE = 0.887, MAE = 0.595), indicating positive out-of-sample predictive relevance for the primary behavioral outcome.

**TABLE 10:** PLSpredict results for endogenous target constructs.

Endogenous target construct	Q^2^_predict	RMSE	MAE
Moral disengagement	0.074	0.970	0.789
Delinquent behavior	0.281	0.887	0.595

Q^2^_predict, predictive relevance statistic from PLSpredict; RMSE, root mean squared error; MAE, mean absolute error.

## 4 Discussion

The present study examined a theory-informed cross-sectional structural model linking youth psychopathic traits, moral disengagement, cognitive empathy, and delinquent behavior among justice-involved adolescents in Saudi social observation centers. The findings showed that youth psychopathic traits were positively associated with both moral disengagement and delinquent behavior. Moral disengagement was also positively associated with delinquent behavior and represented a small but statistically detectable indirect component of the concurrent association between youth psychopathic traits and delinquent behavior. Cognitive empathy showed a negative association with delinquent behavior and statistically qualified the direct association between youth psychopathic traits and delinquent behavior, such that this association was weaker at higher levels of cognitive empathy. These findings are consistent with the study’s social-cognitive moral-regulation framing, in which delinquent behavior is examined in relation to dispositional psychopathy-related traits, moral-cognitive justification processes, and social-cognitive understanding of others’ perspectives.

### 4.1 Psychopathic traits and delinquent behavior

The positive association between youth psychopathic traits and delinquent behavior is consistent with prior evidence linking psychopathy-related traits in juveniles to delinquency and recidivism-related outcomes (Asscher et al., 2011; Geerlings et al., 2020). This association is theoretically coherent because the YPI-S dimensions—grandiose–manipulative, callous–unemotional, and impulsive–irresponsible traits—index interpersonal, affective, and behavioral tendencies that are relevant to delinquency-related risk in adolescence (Colins et al., 2012; Gillen et al., 2019). At the same time, the present results should not be interpreted as showing that youth psychopathic traits provide a complete explanation of delinquent behavior. The effect size for the direct association with delinquent behavior was small (*f*^2^ = 0.056), and the model explained 36.4% of the variance in delinquent behavior through a broader set of concurrent associations involving moral disengagement, cognitive empathy, and their conditional pattern with youth psychopathic traits. This pattern is consistent with multidomain perspectives on juvenile delinquency, which do not treat individual dispositions as sufficient explanations in isolation (Bobbio et al., 2020; Geerlings et al., 2020).

The supplementary dimension-level analysis suggested that the overall psychopathic-traits association with delinquent behavior was not uniformly reflected across the three lower-order dimensions. When grandiose–manipulative, callous–unemotional, and impulsive–irresponsible traits were modeled simultaneously, only grandiose–manipulative traits showed a statistically significant unique direct association with delinquent behavior. This finding is consistent with prior dimension-level evidence suggesting that psychopathic-trait dimensions may differ in their associations with delinquency-related processes. In particular, Kerr et al. (2012) reported a distinct role for grandiose–manipulative traits in deviant peer processes related to adolescent delinquency. However, because Kerr et al. examined peer-influence moderation rather than direct prediction, the present result should be interpreted as a secondary exploratory finding rather than as evidence that grandiose–manipulative traits are generally the strongest predictor of delinquency.

No dimension-specific indirect effect through moral disengagement reached statistical significance. This suggests that the small indirect component observed in the main overall-construct model should not be attributed to a single psychopathic-trait dimension in this sample. It may instead reflect the overall psychopathy-related construct or shared variance among the dimensions. Because the dimension-level analysis was exploratory, cross-sectional, and involved simultaneous modeling of conceptually related dimensions, these findings should be interpreted as secondary clarification rather than as a replacement for the primary overall-construct model.

### 4.2 Moral disengagement as a concurrent moral-cognitive indirect component

Youth psychopathic traits were positively associated with moral disengagement (β = 0.302, *p* < 0.001), and moral disengagement was positively associated with delinquent behavior (β = 0.153, *p* = 0.006). These findings are consistent with Bandura’s social-cognitive theory of moral agency, in which moral self-sanctions can be selectively disengaged when harmful conduct is justified, responsibility is displaced or diffused, consequences are minimized, or victims are blamed or dehumanized (Bandura et al., 1996; Bandura, 2002). The results also align with empirical evidence linking moral disengagement to antisocial behavior, aggression, violence, and transgressive behavior in youth (Hyde et al., 2010; Paciello et al., 2008; Shulman et al., 2011; Luo and Bussey, 2023).

The statistically significant indirect component through moral disengagement indicates that moral disengagement was estimated as a small indirect component of the concurrent association between youth psychopathic traits and delinquent behavior (β = 0.046, *p* = 0.030). This result should be interpreted as a cross-sectional indirect association, not as evidence that youth psychopathic traits temporally produce delinquent behavior through moral disengagement. The indirect estimate was small, and the cross-sectional design does not allow temporal or causal mediation claims. Therefore, the result is best interpreted as evidence that psychopathy-related traits, moral disengagement, and delinquent behavior were structured in a theoretically coherent concurrent pattern. This interpretation is also consistent with prior work suggesting empirical connections between callous–unemotional or psychopathy-related traits and moral disengagement, while cautioning against treating this association as a unidirectional developmental pathway (Muratori et al., 2017; Paciello et al., 2020; Gomez Tabares et al., 2025).

### 4.3 Cognitive empathy and the conditional association between youth psychopathic traits and delinquent behavior

Cognitive empathy was negatively associated with delinquent behavior (β = −0.344, *p* < 0.001) and showed the strongest relative f^2^ contribution among the predictors of delinquent behavior in the structural model (*f*^2^ = 0.146), although its effect size remained slightly below the conventional medium threshold. This finding is consistent with the distinction between cognitive and affective empathy in the Basic Empathy Scale, where cognitive empathy refers to understanding another person’s emotional state or perspective rather than sharing that state affectively (Jolliffe and Farrington, 2006). It also aligns with meta-analytic evidence indicating that cognitive empathy is more strongly related to offending than affective empathy (Jolliffe and Farrington, 2004; van Langen et al., 2014).

The significant interaction between youth psychopathic traits and cognitive empathy (β = −0.175, *p* = 0.007) provides a more specific test of the social-cognitive component of the model. The simple-slope plot indicated that the positive association between youth psychopathic traits and delinquent behavior was strongest when cognitive empathy was low, weaker at the mean level of cognitive empathy, and substantially weaker when cognitive empathy was high. Because cognitive empathy concerns understanding others’ emotional states and perspectives rather than sharing affective states, higher cognitive empathy identified a statistical condition under which psychopathy-related traits were less strongly associated with delinquent behavior. This interpretation is consistent with the theoretical distinction between cognitive and affective empathy in the Basic Empathy Scale and with meta-analytic evidence indicating that cognitive empathy is more closely related to offending than affective empathy (Jolliffe and Farrington, 2004, 2006; van Langen et al., 2014). The finding should not be interpreted as evidence that cognitive empathy causally protects against delinquent behavior. Rather, within this cross-sectional model, cognitive empathy identified a statistical boundary condition in the concurrent association between youth psychopathic traits and delinquent behavior. This finding indicates variation in the strength of the association across levels of cognitive empathy, but it does not establish a protective or causal buffering effect.

### 4.4 Theoretical implications

The findings are consistent with organizing youth psychopathic traits, moral disengagement, and cognitive empathy within a social-cognitive moral-regulation framing. In this model, youth psychopathic traits represented dispositional interpersonal, affective, and behavioral tendencies relevant to delinquency-related risk; moral disengagement represented moral-cognitive justification processes through which harmful conduct may be made less self-sanctioning; and cognitive empathy represented a social-cognitive capacity relevant to understanding others’ emotional states and perspectives (Bandura et al., 1996; Bandura, 2002; Colins et al., 2012; Jolliffe and Farrington, 2006). The results therefore do not position the three constructs as interchangeable predictors. Instead, they indicate that delinquent behavior in this justice-involved sample was associated with distinct but theoretically connected domains.

The pattern of findings is consistent with the view that a purely dispositional account of delinquent behavior may be incomplete. Youth psychopathic traits showed a direct association with delinquent behavior (β = 0.217, *p* < 0.001; *f*^2^ = 0.056), but the pattern of findings also included a moral-cognitive indirect component through moral disengagement (β = 0.046, *p* = 0.030) and a cognitive-empathy interaction (β = −0.175, *p* = 0.007). This pattern is consistent with the theoretical value of examining dispositional risk together with moral self-regulation and social-cognitive understanding.

However, this contribution remains structural and cross-sectional. The present findings can support a theoretically informed account of concurrent associations, but they cannot establish developmental sequencing among youth psychopathic traits, moral disengagement, cognitive empathy, and delinquent behavior.

### 4.5 Practical implications

The findings suggest that research and assessment formulations with justice-involved adolescents may benefit from distinguishing among three individual-level domains rather than treating delinquency-related risk as a single psychological dimension. Youth psychopathic traits may identify dispositional interpersonal, affective, and behavioral tendencies; moral disengagement may indicate the extent to which harmful or rule-violating conduct is accompanied by moral-cognitive justification; and cognitive empathy may provide information about adolescents’ capacity to understand others’ emotional states and perspectives. This distinction is practically relevant because the three constructs showed different roles in the model: youth psychopathic traits were positively associated with delinquent behavior, moral disengagement accounted for a small indirect component of this concurrent association, and cognitive empathy was negatively associated with delinquent behavior while also qualifying the youth psychopathic traits–delinquent behavior association.

These implications should remain limited to assessment formulation and hypothesis generation. The present findings do not show that changing moral disengagement or cognitive empathy would reduce delinquent behavior, nor do they establish intervention effects. However, they suggest that future prevention or rehabilitation research could examine moral-cognitive justification and cognitive perspective understanding alongside psychopathy-related traits. Such work should use longitudinal or intervention designs before drawing conclusions about causal change or program effectiveness.

### 4.6 Limitations

Some limitations restrict the interpretation and generalizability of the findings. First, the cross-sectional design prevents conclusions about temporal ordering or causal mediation. Although the model estimated an indirect component through moral disengagement, the direct, indirect, and interaction paths should be interpreted as concurrent structural associations rather than as evidence of developmental or causal processes.

Second, the study used a consecutive convenience sample of justice-involved adolescents residing in Saudi social observation centers. Although participants were recruited from nine centers across major regions of Saudi Arabia, the sample was not randomly selected from the broader population of justice-involved adolescents. The findings should therefore be generalized cautiously to community adolescents, non-institutionalized justice-involved youth, or adolescents in other legal, institutional, or cultural contexts.

Third, all focal variables were assessed using self-report questionnaires administered at a single time point, and delinquent behavior was reported retrospectively for the preceding 12 months. Common method bias therefore remains a plausible threat to validity despite the procedural safeguards and statistical sensitivity checks reported in the Methods section. The data may also be affected by social desirability, recall limitations, and context-related response pressures. In addition, the delinquency measure did not distinguish whether reported behaviors occurred before admission to the center, during residence in the center, or across both contexts, nor did it differentiate acts by severity or official record status. Accordingly, the delinquency outcome should be interpreted as a self-reported past-year behavioral index rather than as an official or independently verified record of delinquent behavior.

Fourth, although the Arabic item content was prepared through translation, bilingual review, reconciliation, and pilot checking, the present study was not designed as a full independent validation of Arabic versions of the instruments. The measurement-model results provide sample-specific support for the retained indicators in the current structural model, but further psychometric work is needed in larger and more diverse Arabic-speaking justice-involved adolescent samples.

Fifth, several potentially relevant variables were not included in the model, including affective empathy, offense type and severity, prior justice-system involvement, violence-related behavior, length of institutional residence, psychiatric symptoms, substance use, other criminogenic risks, and family or peer factors. Although a supplementary dimension-level analysis was conducted, the primary structural model treated youth psychopathic traits as an overall psychopathy-related construct. Future research should address these issues using longitudinal, multi-informant, and institutionally diverse designs.

### 4.7 Future research

Future research should test the proposed structure using longitudinal designs that can examine temporal ordering among youth psychopathic traits, moral disengagement, cognitive empathy, and delinquent behavior. Such designs would be necessary to evaluate whether moral disengagement functions as a developmental pathway, reciprocal correlate, or concurrent moral-cognitive marker. Multi-informant designs would also strengthen the evidence base by combining adolescent self-report with official records, staff ratings, caregiver reports, or behavioral indicators of delinquency-related outcomes.

Further work should also examine the separate dimensions of youth psychopathic traits rather than relying only on an overall psychopathy-related construct. This is important because prior meta-analytic evidence suggests that the association between psychopathic traits and delinquency differs across psychopathy dimensions (Geerlings et al., 2020). In addition, future studies should test cognitive and affective empathy separately, given evidence that these components may show different associations with offending (Jolliffe and Farrington, 2004; van Langen et al., 2014). Finally, replication in different cultural, legal, and institutional contexts would help determine whether the present pattern is specific to Saudi social observation centers or generalizes to broader justice-involved adolescent populations.

## 5 Conclusion

The present study offers evidence consistent with a theory-informed cross-sectional account of delinquent behavior among justice-involved adolescents. Youth psychopathic traits were positively associated with delinquent behavior and moral disengagement, moral disengagement contributed a small indirect component to the concurrent youth psychopathic traits–delinquent behavior association, and cognitive empathy was negatively associated with delinquent behavior while statistically qualifying the direct association between youth psychopathic traits and delinquent behavior. The model explained 36.4% of the variance in delinquent behavior, while the direct, indirect, and interaction effects remained consistent with a cautious interpretation of concurrent structural associations. These findings are consistent with a social-cognitive moral-regulation account in which delinquent behavior is associated with dispositional psychopathy-related traits, moral-cognitive justification, and social-cognitive understanding. Because the data were cross-sectional and based on self-report, the findings should be interpreted as concurrent structural associations rather than evidence of causal or developmental mechanisms.

## Data Availability

The datasets presented in this article are not readily available because the data involve minors residing in social observation centers and are subject to ethical and institutional restrictions. Public deposition of the dataset was not permitted by the ethics approval and participant consent. Access, if granted, is limited to de-identified analytic materials and is subject to approval by the relevant ethical and institutional authorities. Participant-identifiable data will not be shared. Requests to access the datasets should be directed to almailabi@iu.edu.sa.
